# Targeting regulated cell death pathways in lung cancer: mechanisms, therapeutic strategies, and clinical translation

**DOI:** 10.3389/fimmu.2026.1703943

**Published:** 2026-02-18

**Authors:** Fangsu Xue, Jiacheng Sun, Jitai Zhang, Yuntian Shen

**Affiliations:** 1 Department of Respiration, Binhai County People’s Hospital, Yancheng, Jiangsu, China; 2 Jiangsu Key Laboratory of Tissue Engineering and Neuroregeneration, Key Laboratory of Neuroregeneration of Ministry of Education, Co-Innovation Center of Neuroregeneration, Medical School of Nantong University, Affiliated Hospital of Nantong University, Nantong University, Nantong, Jiangsu, China

**Keywords:** drug resistance mechanisms, lung cancer, regulated cell death, targeted therapy, tumor microenvironment

## Abstract

Lung cancer is the leading cause of global cancer mortality, with treatment efficacy limited by high heterogeneity, drug resistance, and an immunosuppressive tumor microenvironment. Focusing primarily on non-small cell lung cancer (NSCLC), this review systematically analyzes eight key regulated cell death (RCD) pathways in lung cancer. These pathways are apoptosis, autophagy, necroptosis, ferroptosis, cuproptosis, pyroptosis, immunogenic cell death (ICD), and lysosome-dependent cell death (LDCD). Mechanistic dissection reveals complex crosstalk and a dynamic equilibrium among these pathways. For instance, apoptosis escape via EGFR/PI3K/Akt/mTOR signaling promotes survival, while autophagy exhibits a context-dependent dual role regulated by factors such as RBBP4 and the AURKA-CXCL5 axis. Importantly, several RCD pathways exert potent immunomodulatory functions. Necroptosis activates T cells by releasing damage-associated molecular patterns (DAMPs), while ferroptosis enhances NK cell cytotoxicity through GPX4 inactivation. Regarding therapeutic advances, synergistic strategies show promise, such as berberine with EGFR-TKIs inducing apoptosis via EGFR degradation, and (-)-Guaiol triggering ICD to synergize with PD-1/PD-L1 inhibitors. Novel inducers, including Auranofin (ferroptosis), TMEM100 agonists (necroptosis), and cuproptosis nanomedicines (e.g., DE-Cu_4_O_3_ NPs), demonstrate preclinical potential. Prognostic models based on RCD-related genes (e.g., LDCD signatures) can predict immune features and response to immune checkpoint inhibitors (ICIs). However, clinical translation faces bottlenecks, including intricate pathway crosstalk, difficulties in remodeling the immunosuppressive niche, low ICI response in EGFR-mutant patients, and a lack of standardized biomarkers and optimized delivery systems. Future research should prioritize coordinated targeting of multiple death pathways, utilize advanced computational tools integrated with multi-omics data to decipher RCD network complexity and optimize treatment prediction, and strengthen interdisciplinary translational efforts. Ultimately, a deep understanding of the RCD network paves the way for a paradigm shift toward precision therapy in lung cancer.

## 1 Introduction

Lung cancer is the leading cause of global cancer-related deaths. Non-small cell lung cancer (NSCLC) accounts for approximately 85% of these cases. Conventional treatments like surgery, radiotherapy, and chemotherapy have significant limitations. Targeted therapies and immunotherapy improve survival yet still face challenges like drug resistance and variable efficacy among individuals (1, 2). The high heterogeneity of lung cancer demands deep understanding of its molecular mechanisms. Central to tumorigenesis is dysregulation of cell death pathways. Recent discoveries in RCD pathways offer new perspectives for overcoming resistance. Unlike traditional cytotoxic chemotherapy, targeting these pathways enables precise intervention in cell fate decisions. This approach can selectively induce tumor cell death and remodel the immune microenvironment. Notably, these pathways exhibit a dynamic dual nature in lung cancer. They can suppress tumor progression but can also be co-opted by cancer cells for survival. For instance, evasion of apoptosis is central to resistance in EGFR-mutant lung cancer. EGFR signaling activates the PI3K/Akt/mTOR axis. This suppresses pro-apoptotic Bax and upregulates anti-apoptotic Bcl-2, directly promoting cell survival (3, 4). Furthermore, low TMEM100 expression inhibits apoptosis via the same pathway. It also induces protective autophagy, accelerating TNM stage progression (5). Additionally, osimertinib-resistant cells employ metabolic reprogramming. They enhance glycolysis and glutamine uptake to sustain mitochondrial function, further resisting apoptosis (6). This imbalance in dynamic equilibrium causes compensatory resistance during single-pathway targeting. Therefore, understanding the complex RCD network is crucial for enhancing therapeutic efficacy.

Specific regulated cell death pathways with immunomodulatory functions represent core strategies for reversing tumor microenvironment immunosuppression. In necroptosis and immunogenic cell death research, RIPK1/RIPK3/MLKL-mediated necroptosis triggers antitumor immunity by releasing damage-associated molecular patterns (DAMPs) through plasma membrane rupture. The natural compound (-)-Guaiol relies on this pathway to induce immunogenic cell death, activating dendritic cells and enhancing T-cell infiltration (7). Ferroptosis studies reveal that lipid reactive oxygen species (ROS) accumulation caused by GPX4 inactivation is particularly prominent in EGFR-mutant lung cancer. The drug Auranofin induces ferroptosis by inhibiting thioredoxin reductase 1 (TrxR1) and releases DAMPs, thereby enhancing natural killer (NK) cell cytotoxicity (6, 8, 9). Cuproptosis research further expands metabolic intervention potential. Copper ion homeostasis imbalance disrupts mitochondrial tricarboxylic acid (TCA) cycle activity, inducing cell death. Dysregulation of DLAT/DLST genes promotes lung cancer metastasis, while LIPT1 suppresses tumor progression by downregulating the copper chaperone ATOX1 (10, 11). Pyroptosis activates the NLRP3 inflammasome via Gasdermin proteins, releasing IL-1β/IL-18 (12). The natural compound Sophflarine A activates the NLRP3/caspase-1/GSDMD axis through ROS signaling, synergistically inducing autophagy and pyroptosis (13–15). Furthermore, LDCD releases cathepsins through lysosomal membrane permeabilization (LMP), activating downstream apoptotic or necrotic pathways. Disrupting lysosomal Ca²^+^ balance via the MCOLN3 channel blocks autophagic flux and promotes cell death (2, 16). These mechanisms establish a theoretical foundation for developing novel immunotherapies targeting the tumor microenvironment.

Based on these mechanisms, multi-pathway interventions show potential for overcoming drug resistance. In apoptosis-autophagy synergy, berberine plus icotinib degrades EGFR and accumulates ROS to reverse apoptosis resistance (17). Baicalin blocks autophagic flux by activating MCOLN3 channels, inducing mitochondrial apoptosis (16). For immunotherapy, (-)-Guaiol triggers immunogenic cell death (ICD). Combining it with PD-1/PD-L1 inhibitors synergistically activates T-cell responses (18). Dynamic monitoring of sPD-L1 and exoPD-L1 levels helps predict efficacy (19). Novel death pathway strategies include Auranofin, a ferroptosis inducer acting through metabolic disruption, and TMEM100 activation, which promotes necroptosis via gene regulation. Both enhance antitumor immunity (5, 8). AI-driven precision therapy holds great promise. Prognostic models based on LDCD-related genes (e.g., LAMP1, CTSB) predict immune microenvironment features and immunotherapy response. This provides a molecular basis for individualized treatment (2, 20). Collectively, these complementary approaches represent a promising frontier in overcoming therapeutic resistance and improving cancer treatment outcomes.

Lung cancer therapy faces three critical challenges. First, the complexity of pathway interaction networks requires dual-pathway inducers. For example, autophagy inhibition enhances TMEM100-mediated necroptosis (5, 16). Second, reshaping the immunosuppressive microenvironment remains difficult. EGFR-mutant lung cancers respond poorly to immune checkpoint inhibitors (ICIs). Combining VEGF inhibitors with chemotherapy yields only modest survival improvements (16). Third, clinical translation encounters significant barriers. These include targeted delivery system development, validation of LDCD scoring models, and standardization of PD-L1 testing (2, 21). Future research must therefore focus on novel mechanisms like methionine restriction-induced cell death. Multi-omics guided coordination of death pathways is essential. Artificial intelligence should integrate single-cell and spatial transcriptomic data to optimize treatment prediction. These efforts will drive a paradigm shift in precision lung cancer therapy. Collaborative advances will overcome existing barriers and enhance patient survival outcomes.

Therefore, to bridge these mechanistic insights with clinical translation, this review is structured along the following translational logic. First, we dissect the molecular mechanisms and dual roles of eight key RCD pathways—apoptosis, autophagy, necroptosis, ferroptosis, cuproptosis, pyroptosis, ICD, and lysosome-dependent cell death—in tumor progression and drug resistance, with a primary focus on NSCLC while noting subtype-specific vulnerabilities where evidence is clear (e.g., the unique sensitivity of EGFR-mutant tumors to ferroptosis). Second, we synthesize emerging therapeutic strategies that target these pathways individually or in combination, with a focus on reversing immunotherapy resistance. Third, we evaluate the current landscape of predictive biomarkers, highlighting the integration of conventional markers (e.g., PD-L1, tumor mutational burden) with novel RCD-based signatures for patient stratification. Finally, we present future perspectives, emphasizing the need for synergistic multi-pathway targeting, advanced computational approaches to decipher RCD network complexity, and overcoming translational bottlenecks to usher in a new era of precision oncology for lung cancer.

## 2 Mechanisms of regulated cell death in lung cancer

**Figure 1:**
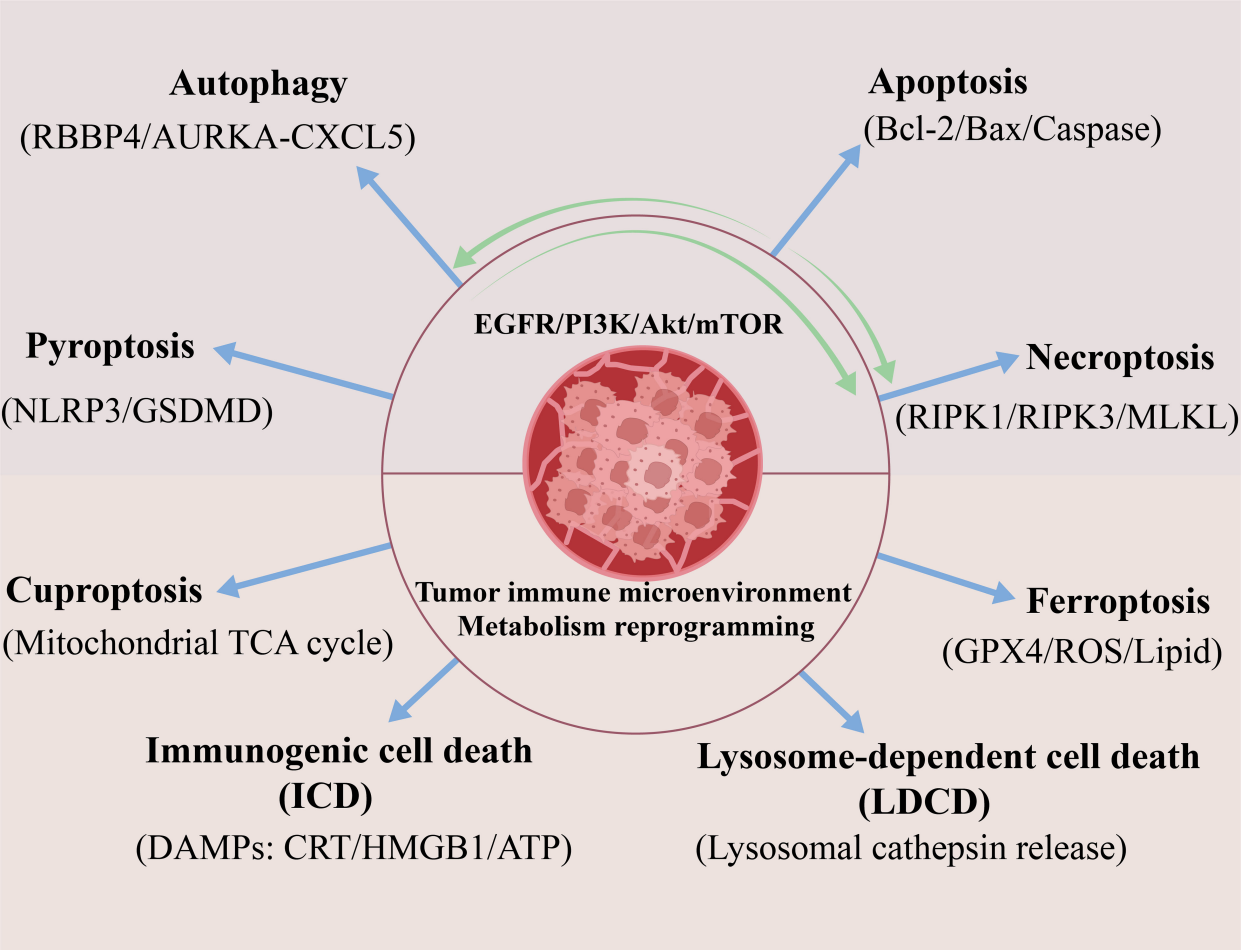
The interaction network of RCD pathways in NSCLC. This figure summarizes eight major RCD pathways that drive lung cancer progression and therapy resistance. These include: apoptosis (via the EGFR/PI3K/Akt/mTOR axis), autophagy (dual role regulated by RBBP4/AURKA-CXCL5), necroptosis (mediated by RIPK1/RIPK3/MLKL), ferroptosis (triggered by GPX4 inactivation and lipid ROS accumulation), cuproptosis (driven by disruption of mitochondrial TCA cycle), pyroptosis (via the NLRP3/GSDMD pathway), immunogenic cell death (ICD; characterized by release of DAMPs such as CRT, HMGB1, and ATP, and lysosome-dependent cell death (LDCD; involving lysosomal cathepsin release). Dynamic crosstalk exists among these pathways; for instance, autophagy inhibition enhances necroptosis, while ferroptosis synergizes with ICD to activate immune responses. Collectively, dysregulation of these pathways not only mediates drug resistance (e.g., apoptosis escape in EGFR-mutant tumors) but also shapes an immunosuppressive tumor microenvironment.

The high heterogeneity of lung cancer and its core challenge of therapeutic resistance urgently demand in-depth analysis of the complex mechanisms underlying RCD pathways. These pathways, including apoptosis, autophagy, necroptosis, ferroptosis, cuproptosis, pyroptosis, immunogenic cell death, and LDCD, play dual roles in tumorigenesis, progression, and therapeutic response. Cancer cells can hijack these pathways to evade death, yet they also represent critical targets for precision intervention. This section will systematically explore the molecular mechanisms and regulatory networks of eight key RCD pathways in lung cancer (**Figure 1**). It will also examine their central roles in overcoming drug resistance and remodeling the tumor microenvironment.

### 2.1 Apoptosis

In lung cancer, apoptosis serves as the primary programmed cell death mechanism. It operates mainly through caspase cascade reactions. The underlying pathways involve mitochondrial and death receptor signaling. However, tumor cells frequently evade this process. For instance, EGFR mutations activate the PI3K/Akt/mTOR signaling axis. This activation suppresses pro-apoptotic proteins like Bax. Simultaneously, it upregulates anti-apoptotic factors such as Bcl-2, thereby promoting tumor survival (3, 4). Similarly, the transmembrane protein TMEM100 functions as a tumor suppressor. Its low expression in NSCLC activates the PI3K/AKT pathway. This activation inhibits apoptosis while inducing autophagy. Ultimately, it leads to TNM stage progression and poor prognosis (5). Critically, evasion of apoptosis represents a core mechanism of lung cancer drug resistance. For example, osimertinib-resistant cells exhibit metabolic reprogramming. They enhance glycolysis and glutamine uptake. This adaptation sustains mitochondrial respiratory function and resists apoptosis (6). To address this challenge, natural compounds show promise. Baicalin activates lysosomal channel MCOLN1, disrupting calcium homeostasis. This triggers mitochondrial membrane potential collapse and ROS accumulation, leading to caspase-dependent apoptosis (16). Berberine combined with icotinib promotes EGFR degradation and ROS accumulation. This combination synergistically overcomes apoptosis resistance in EGFR wild-type lung cancer (17). Thus, targeting key nodes in apoptosis pathways is an effective strategy. These nodes include EGFR/PI3K signaling and metabolic adaptation. Future research must deeply explore the regulatory networks of these targets. It must also develop more precise combination strategies. This will overcome apoptosis resistance and improve patient outcomes.

### 2.2 Autophagy

Autophagy plays a context-dependent dual role in lung cancer, functioning as both a pro-survival mechanism and a potential cell death pathway. Under metabolic stress, it maintains cellular homeostasis by clearing damaged organelles and proteins, thereby promoting tumor cell survival (22, 23). However, excessive or dysregulated autophagy can trigger autophagic cell death. The specific outcome is governed by a network of regulatory molecules and pathways, often involving key nodes in lung cancer signaling.

Key regulatory genes critically influence this balance. For instance, RBBP4 overexpression suppresses autophagic activity and is associated with poor prognosis, while its knockdown induces autophagic cell death by inhibiting the PI3K/Akt/mTOR pathway, significantly suppressing tumor growth *in vitro* and *in vivo* (24). This highlights that modulating core pathways like PI3K/Akt/mTOR can switch autophagy from a pro-survival to a pro-death process. Similarly, the AURKA-CXCL5 signaling axis regulates autophagy and impacts radiotherapy sensitivity in NSCLC. Inhibiting this axis enhances radiotherapy-induced cytotoxic autophagy, helping to overcome radioresistance (25). Therapeutic interventions can exploit this duality by targeting specific components. The natural compound baicalin activates the lysosomal cation channel MCOLN3, disrupting Ca²^+^ homeostasis and lysosomal function. This blockade of autophagic flux, accompanied by mitochondrial membrane depolarization and ROS accumulation, leads to autophagy-dependent apoptosis (16). Conversely, the transmembrane protein TMEM100 induces protective autophagy by suppressing the PI3K/AKT pathway. Combining strategies that target such specific regulators, for example, using TMEM100 alongside autophagy inhibitors, can synergistically promote cancer cell death (5, 26). Therefore, molecules like MCOLN3, RBBP4, and the AURKA-CXCL5 axis are not only key determinants of autophagy’s dual role but also represent critical therapeutic targets. Their targeted modulation offers a promising strategy to overcome treatment resistance by precisely steering autophagy toward a tumor-suppressive outcome. A deeper understanding of these regulatory networks is essential for developing the next generation of lung cancer therapies.

### 2.3 Necroptosis

Necroptosis is a programmed necrotic process mediated by receptor-interacting protein kinases RIPK1/RIPK3 and mixed lineage kinase domain-like protein (MLKL) (27). It features plasma membrane rupture, release of cellular contents, and strong inflammatory responses. This lytic, inflammatory outcome is shared with pyroptosis, but necroptosis is typically initiated when apoptotic caspases are inhibited, representing an alternative death pathway. In NSCLC, studies implicate necroptosis in therapy response; for instance, the natural product (-)-Guaiol exerts antitumor effects partially through necroptosis-associated DAMP release, enhancing anti-tumor immunity (7). Furthermore, the dysregulation of genes related to other death pathways, such as lysosome-dependent cell death, can stratify patient risk and predict immune microenvironment features, indirectly underscoring the clinical relevance of necrotic processes (2, 28). The potent immunogenicity of necroptosis makes it an attractive target for overcoming immunotherapy resistance. However, its clinical translation faces significant hurdles, primarily the challenge of achieving tumor-selective activation of the RIPK1/RIPK3/MLKL axis to avoid uncontrolled systemic inflammation. Current strategies are preclinical, focusing on small-molecule inducers or leveraging endogenous regulators like TMEM100 (5). The most viable path forward involves rational combination therapies, such as pairing necroptosis inducers with ICIs to convert lytic cell death into a sustained antitumor immune response. Future clinical development will depend on identifying reliable biomarkers to select patients most likely to benefit from such interventions.

### 2.4 Ferroptosis

Ferroptosis is an iron-dependent form of regulated cell death driven by the accumulation of lipid peroxides following glutathione peroxidase 4 (GPX4) inactivation. This process leads to oxidative damage of cell membranes (6, 29, 30). This execution via metabolic catastrophe—specifically, iron-dependent lipid peroxidation—distinguishes it from protease-driven apoptosis or pore-forming necroptosis/pyroptosis. Its relevance in lung cancer is highlighted by the unique vulnerability of EGFR-mutant, as opposed to wild-type NSCLC cells, which undergo metabolic reprogramming that heightens sensitivity to ferroptosis induction (6). Agents like Auranofin induce ferroptosis by inhibiting thioredoxin reductase 1 (TrxR1), leading to profound ROS accumulation (8). This oxidative stress drives both the execution of ferroptosis via lipid peroxidation and the promotion of an immunogenic tumor microenvironment. The rupture of the plasma membrane during ferroptosis releases DAMPs, such as high-mobility group box 1 (HMGB1) and ATP. These signals activate antigen-presenting cells (e.g., dendritic cells), promote their maturation and migration to lymph nodes, and enhance the tumoricidal function of NK cells, thereby converting immunologically “cold” tumors into “hot” ones (8, 31). This provides a strong rationale for combining ferroptosis inducers with immunotherapy. The appeal of targeting ferroptosis lies in its ability to eliminate apoptosis-resistant, metabolically active tumors. Translating this promise to the clinic requires overcoming key challenges, including the development of specific and bioavailable ferroptosis inducers beyond preclinical tool compounds. The context-dependent nature of its immunogenicity also necessitates careful patient stratification. Near-term strategies involve repurposing existing drugs with ferroptosis-inducing properties (e.g., sulfasalazine) or combining standard therapies (e.g., radiotherapy, certain chemotherapies) that can sensitize tumors to ferroptosis. Validating biomarkers of lipid peroxidation in patient samples will be crucial for guiding these emerging clinical trials.

### 2.5 Cuproptosis

Cuproptosis represents a copper-dependent form of cell death. Its core mechanism involves disrupting mitochondrial respiration and the TCA cycle (32). This ultimately causes cellular metabolic collapse. It shares with ferroptosis the concept of exploiting metal ion homeostasis but is mechanistically distinct, focusing on mitochondrial protein aggregation and TCA cycle disruption rather than lipid peroxidation. In NSCLC, abnormal expression of cuproptosis-related genes significantly impacts patient prognosis and metastasis. Specifically, dysregulation of dihydrolipoamide S-acetyltransferase (DLAT) and dihydrolipoamide S-succinyltransferase (DLST) promotes tumor metastasis. Their expression levels negatively correlate with patient survival rates (11). Furthermore, functional studies indicate that elevated expression of lipoyltransferase 1 (LIPT1) is associated with a favorable prognosis in NSCLC. Overexpression of LIPT1 was shown to inhibit tumor cell growth and promote apoptosis, concomitant with the downregulation of the copper chaperone ATOX1, suggesting a role for LIPT1 in impeding NSCLC progression through modulation of copper homeostasis (10). These genes primarily drive copper ion accumulation and induce proteotoxic stress. They achieve this by regulating mitochondrial depolarization such as through NDUFA4L2 and TCA cycle function. This process thereby mediates cell death. Beyond key genes, cuproptosis-related molecular markers provide essential tools for NSCLC management. For instance, FDX1 autoantibodies and specific long non-coding RNA (lncRNA) signatures accurately predict NSCLC prognosis. Studies have constructed a risk model based on six lncRNAs including AC104088.1 and LUCAT1. Patients in the high-risk group showed significantly shorter overall survival (33). Notably, mitochondrial depolarization-related genes such as DCN and PTHLH exhibit synergy with cuproptosis. Their risk scores independently predict patient response to immunotherapy and chemotherapy sensitivity (34). However, external factors like COVID-19 infection exacerbate abnormal expression of cuproptosis-related lncRNAs such as HHLA3-AS1. This further worsens survival outcomes for NSCLC patients (35, 36). Regarding therapeutic strategies, nanotherapies targeting cuproptosis show promise. For example, diethyldithiocarbamate-Cu_4_O_3_ nanocomposites (DE-Cu_4_O_3_ NPs) selectively kill lung cancer stem cells. They do this by inducing mitochondrial dysfunction and telomerase inactivation through inhibiting TERT/TRF1. Importantly, they demonstrate no systemic toxicity (37). Simultaneously, FDX1 autoantibody-based detection offers a new avenue for early diagnosis. This technique shows good diagnostic efficacy in distinguishing NSCLC from benign pulmonary nodules, achieving an area under the curve (AUC) of 0.806 (38). In summary, a deeper understanding of cuproptosis regulatory networks in NSCLC and their interaction with the immune microenvironment is crucial. This understanding will lay the essential foundation for developing more effective prognostic models and targeted therapeutic strategies.

### 2.6 Pyroptosis

Pyroptosis represents a Gasdermin-mediated programmed cell death (39). It regulates tumor progression in NSCLC through the inflammasome pathway. Mechanistically, studies show cucurbitacin B (CuB) directly binds Toll-like receptor 4 (TLR4). This binding activates the NLRP3 inflammasome and cleaves Gasdermin D (GSDMD) into N-terminal and C-terminal fragments. Consequently, it triggers plasma membrane pore formation and IL-1β/IL-18 release, thereby inhibiting NSCLC growth (13). These released cytokines are pivotal for reversing local immunosuppression. IL-1β promotes dendritic cell maturation and the differentiation of CD4^+^ T cells into a T-helper 1 (Th1) phenotype, which is critical for activating cytotoxic CD8^+^ T cells (40, 41). IL-18 synergizes with IL-12 to enhance interferon-γ (IFN-γ) production, thereby boosting the antitumor activity of CD8^+^ T cells and NK cells (42, 43). In contrast to the immunologically silent nature of apoptosis, pyroptosis creates a potent pro-inflammatory microenvironment through lytic cell death and cytokine release, a feature it shares with necroptosis but executes via a distinct inflammasome-Gasdermin axis. Importantly, this pathway is enhanced by mitochondrial ROS and calcium ion (Ca²^+^) release. TLR4 silencing significantly suppresses pyroptosis (13). Therefore, the TLR4/NLRP3/GSDMD axis is confirmed as a core signaling hub regulating pyroptosis in lung cancer. From a therapeutic perspective, natural compounds targeting ROS-dependent pyroptosis pathways show promise. For instance, sophflarine A increases ROS generation to activate the NLRP3/caspase-1/GSDMD pathway. Simultaneously, it blocks the PI3K/AKT/mTOR signal to induce autophagy, synergistically inhibiting NSCLC proliferation (14). Similarly, reniformin A also directly binds TLR4 to promote NLRP3 inflammasome assembly. Its pro-pyroptotic effect is completely reversed by TLR4 knockout (44). These findings collectively highlight the critical value of ROS and natural compounds in targeting pyroptosis pathways. Clinically, pyroptosis closely relates to the tumor microenvironment and drug resistance. Specifically, in EGFR-T790M mutation-resistant models, shikonin degrades COX-2 protein. This degradation induces caspase-3/GSDME-dependent pyroptosis, reversing platinum-based drug resistance (45). In contrast, lncRNA16 adsorbs miR-1827 to upregulate MBD3. This upregulation suppresses GSDME expression, weakening cisplatin-induced pyroptosis (46). Moreover, polymorphisms in pyroptosis-related genes (like GSDMB, AIM2) significantly associate with NSCLC risk. High expression of ASC/CASP1 predicts a favorable prognosis (47–49). In summary, the interaction between pyroptosis pathways and the tumor immune microenvironment, along with chemotherapy resistance, offers new perspectives for lung cancer treatment. These studies deepen the understanding of pyroptosis mechanisms in NSCLC cells. Furthermore, they lay an important foundation for developing novel therapeutic strategies targeting this pathway.

### 2.7 Immunogenic cell death

ICD is a functionally defined process that primes an adaptive antitumor immune response through the spatiotemporally coordinated release of specific DAMPs, including calreticulin (CRT) exposure on the cell surface and the extracellular secretion of HMGB1 and ATP (50). These signals collectively drive dendritic cell maturation and the initiation of tumor-specific T-cell responses (51). ICD represents a functional state—often arising from specific forms of apoptosis, necroptosis, or other stresses—but is distinguished by its orchestrated immunogenic output, setting it apart from passive inflammatory release. In lung cancer, diverse agents induce ICD via distinct primary mechanisms: (-)-Guaiol triggers DAMP release by coordinating apoptosis and autophagy pathways (51); an iridium (III) complex induces endoplasmic reticulum stress and ROS accumulation, elevating HMGB1 and ATP secretion (52, 53)); sea hare hydrolysates (SHH) inhibit STAT3 to induce mitochondrial dysfunction and caspase-1-mediated death (54); and dihydroartemisinin (DHA) combined with cisplatin amplifies ICD through the ROS-ER stress-JNK/p38 MAPK cascade (55). These diverse mechanisms converge on remodeling the tumor immune microenvironment, providing a crucial synergistic foundation for ICIs and opening new combination strategies for immunotherapy. While ICD induction is a compelling preclinical strategy to convert immunologically “cold” tumors, its clinical translation faces defined challenges. A primary bottleneck is the lack of standardized, validated pharmacodynamic biomarkers (e.g., plasma HMGB1, extracellular ATP) to reliably monitor ICD induction in patients during trials. Furthermore, most potent ICD inducers, including many natural compounds and metal complexes, remain in the preclinical or early exploratory stage, necessitating the development of clinically suitable agents with optimal pharmacokinetics and safety profiles. The most promising and direct translational pathway is the rational combination of ICD-inducing therapies with immune checkpoint blockade. Early-phase clinical trials are actively exploring this paradigm, such as combining certain chemotherapies known to elicit ICD (e.g., anthracyclines, oxaliplatin) with anti-PD-1/PD-L1 antibodies. Success in these trials could establish pharmacological ICD induction as a key strategy to overcome primary and acquired resistance to immunotherapy in lung cancer.

### 2.8 Lysosome-dependent cell death

Unlike classical cell death pathways like apoptosis and necroptosis, LDCD plays a key role in lung cancer progression. It triggers cathepsin release via lysosomal membrane permeabilization (LMP). This activates downstream apoptotic or necrotic pathways (56). Specifically, LMP causes leakage of lysosomal contents, such as cathepsins B/D, into the cytosol. This leads to mitochondrial membrane potential collapse and reactive oxygen species accumulation. Ultimately, it induces caspase-dependent apoptosis or programmed necrosis (2). Studies show aberrant expression of LDCD-related genes, like MCOLN3, promotes lung cancer cell death. It disrupts lysosomal Ca²^+^ balance and pH homeostasis and blocks autophagic flux (16). Notably, an LDCD prognostic scoring model was built using single-cell transcriptome analysis. It integrates 12 core genes, including LAMP1 and CTSB. This model effectively stratifies the immune microenvironment features of NSCLC patients. The high-risk group shows increased monocyte infiltration and enhanced cell communication. It also correlates significantly with poorer survival outcomes (2). This model predicts patient response to ICIs. It also provides a molecular stratification basis for precision therapy targeting the lysosomal pathway. Therefore, deeper exploration of LDCD mechanisms advances understanding of lung cancer cell death pathways. It also opens new directions for developing personalized treatment strategies. This highlights the vast potential for translating basic mechanisms into clinical applications.

### 2.9 Comparative synthesis of RCD pathways: a mechanistic and immunogenic framework

The evidence and discussion presented in this review focus primarily on NSCLC, with an emphasis on adenocarcinoma. An important consideration is that the activity and therapeutic potential of RCD pathways are likely to vary between different lung cancer subtypes, reflecting their distinct pathological backgrounds. For example, the unique metabolic state of EGFR-mutant NSCLC increases its sensitivity to ferroptosis, while small cell lung cancer (SCLC) may harbor a differently configured RCD network due to its separate oncogenic drivers. Future work to define these subtype-specific profiles will be key to advancing precision therapy.

A comparative analysis of the eight major RCD pathways indicates they can be categorized according to two principal features: their capacity to stimulate an immune response (immunogenic output) and the core biochemical mechanism that executes cell death. These features determine their therapeutic potential. In terms of immunogenicity, a clear hierarchy exists. ICD, necroptosis, and pyroptosis are most potent at activating anti-tumor immunity, making them prime candidates for combination with ICIs. ICD works through the orchestrated release of specific DAMPs. In contrast, necroptosis and pyroptosis cause inflammatory plasma membrane rupture, passively releasing intracellular contents and cytokines to recruit and activate immune cells. Critically, these processes reverse local immunosuppression. They deliver key signals like CRT, HMGB1, ATP, IL-1β, and IL-18. These signals promote dendritic cell maturation, drive Th1 and cytotoxic T-cell differentiation, and enhance NK cell activity (40, 41). This converts immunologically “cold” tumors into “hot” ones. In stark contrast, apoptosis is typically immunologically silent. Ferroptosis and cuproptosis can be contextually immunogenic, mainly through secondary stress signals. Autophagy and lysosome-dependent cell death exert only indirect immunogenic effects, depending on the terminal death pathway they engage.

A pivotal factor underlying the crosstalk among multiple RCD pathways is ROS. It functions not merely as a byproduct but as a central stress amplifier and shared node that can initiate, modulate, and link distinct death modalities. Mitochondrial ROS contributes to intrinsic apoptosis and can activate the NLRP3 inflammasome, thereby promoting pyroptosis. In autophagy, ROS levels critically influence its outcome. Moderate increases may induce cytoprotective autophagy, whereas excessive ROS can trigger autophagic cell death or facilitate a shift toward other lethal pathways. Most directly, the execution of ferroptosis is driven by iron-dependent lipid peroxidation, a process fundamentally reliant on ROS. This common dependency on redox imbalance creates a network in which the induction of one RCD, for example, ferroptosis, can elevate cellular ROS to a threshold that sensitizes cells to, or directly triggers, another, such as pyroptosis. Consequently, ROS represents a key mechanistic link that mediates synergistic and regulatory interactions within the RCD network, offering a strategic target for rational combination therapies.

Beyond immunogenicity, these pathways use distinct biochemical principles to execute cell death. They fall into several mechanistic classes. Protease-dependent pathways include apoptosis (via caspases) and pyroptosis (via inflammatory caspases and gasdermins). Membrane disruption-dependent pathways include necroptosis (via MLKL pores) and pyroptosis (via gasdermin pores). Metabolic catastrophe-dependent pathways include ferroptosis (via lipid peroxidation) and cuproptosis (via mitochondrial dysfunction). Autophagy is a self-digestion-dependent pathway. This mechanistic diversity is a therapeutic advantage. Cancer cells resistant to one form of death, such as caspase-inhibited apoptosis, may remain vulnerable to another, such as necroptosis targeting membrane integrity or ferroptosis targeting metabolic pathways.

This integrative view leads to a central therapeutic insight. The diversity of RCD pathways provides a multi-pronged strategy to attack tumors. Choosing which pathway(s) to target should be informed by the specific genetic, metabolic, and immunological context of the individual tumor. A promising approach is the rational combination of agents that induce immunogenic death (e.g., ICD or pyroptosis inducers) to remodel the tumor immune microenvironment, together with agents that exploit distinct metabolic vulnerabilities (e.g., ferroptosis inducers) to directly kill resistant cell populations. The next section examines the emerging therapeutic strategies that stem from this integrated understanding.

## 3 Targeting regulated cell death in lung cancer: from mechanistic insights to therapeutic integration

The preceding comparative analysis of RCD pathways delineates a spectrum of immunogenic potential, targetability, and utility in overcoming specific resistance mechanisms. This functional taxonomy necessitates a therapeutic paradigm shift from isolated pathway modulation toward integrated strategies that either co-activate complementary death modalities or rationally combine RCD inducers with immunotherapy. Building on this foundation, the present section synthesizes the translational landscape, structured to reflect this evolving logic: from foundational single-pathway strategies to synergistic multi-pathway interventions and immune-remodeling combinations, concluding with approaches designed to subvert acquired resistance. Our goal is to outline a coherent framework for designing precision therapies that leverage the distinct yet interconnected vulnerabilities of lung cancer cells, as systematized in **Table 1**.

**Table 1:** Treatment strategies for NSCLC targeting RCD.

Drugs	Treatment mechanism	Targeted RCD	Ref.
Baicalin	MCOLN3 activation and ROS accumulation	Autophagy-related apoptosis	(Dong et al.)
Berberine combined with icotinib	EGFR degradation and ROS accumulation	Apoptosis	(Chen et al.)
Aloe-emodin	MAPK signaling pathway and Akt/mTOR pathway	Apoptosis	(Shen et al.)
Vorinostat combined with gefitinib	HSP90/EGFR/MET/AKT	Apoptosis	(Park et al.)
Exopolysaccharides from the polar yeast Cryptococcus heimaeyensis	ROS/p38/ROS/ERK	Autophagy	(Hao et al.)
Polyphyllin VI	PI3K/AKT/mTOR	Autophagy and apoptosis	(Teng et al.)
Small molecule agonists targeting receptor-interacting protein kinase	Destroying lysosomal function and calcium ion balance	Necroptosis	(Garnique et al.)
(-)-Guaiol	Damage-associated molecular patterns (DAMPs) release	Immunogenic cell death and necroptosis	(Yang et al.)
Auranofin	TrxR1 inhibition and ROS accumulation; DAMPs release and dendritic cell activation	Ferroptosis	(Freire Boullosa et al.)
DE-Cu_4_O_3_ NPs	Mitochondrial dysfunction and telomerase inactivation	Cuproptosis	(Abu-Serie and Blasco)
Cucurbitacin B	TLR4/NLRP3/GSDMD/IL-1β	Pyroptosis	(Yuan et al.)
Sophflarine A	NLRP3/caspase-1/GSDMD	Pyroptosis and autophagy	(Luo et al.)
Reniformin A	TLR4/NLRP3	Pyroptosis	(Zhu et al.)
Shikonin	COX-2/caspase-1/GSDME	Pyroptosis	(Cao et al.)
Iridium (III) complex	Endoplasmic Reticulum stress and ROS accumulation	Immunogenic cell death	(Wang et al.)
sea hare hydrolysates (SHH)	STAT3/ROS/caspase-1	Immunogenic cell death and pyroptosis and necroptosis	(Nyiramana et al.)
Dihydroartemisinin (DHA) combined with cisplatin	ROS-endoplasmic reticulum stress-JNK/p38 MAPK cascade	Immunogenic cell death	(Ni et al.)
Evodiamine combined with cisplatin	Epigenetic regulation	Autophagy	(Panda et al.)

### 3.1 Targeted cell death strategies for lung cancer treatment

Apoptosis is the core form of programmed cell death. It serves as a crucial target for lung cancer therapy. Small molecule inhibitors exert antitumor effects by targeting apoptosis regulatory pathways. Aloe-emodin significantly inhibits proliferation in NSCLC cell lines, such as A549 and H1299 (57). It activates the MAPK signaling pathway and inhibits the Akt/mTOR pathway. This leads to mitochondrial membrane potential loss and ROS accumulation. Consequently, it initiates caspase-dependent apoptosis. Berberine combined with EGFR tyrosine kinase inhibitors, like icotinib, induces autophagy-dependent apoptosis (17). This combination promotes autophagic degradation of the EGFR protein. It also increases intracellular ROS levels. This effectively overcomes resistance in EGFR wild-type/K-RAS mutant NSCLC cells, including H460 and H1299. Combination therapeutic strategies significantly enhance apoptotic effects through synergistic mechanisms. For example, the histone deacetylase inhibitor (HDACi) vorinostat combined with gefitinib substantially increases apoptosis rates in resistant NSCLC cells, such as PC9 and PC9GR (58). This synergy relies on ROS-triggered caspase cascade activation. It causes cleavage of heat shock protein 90 (HSP90) and degradation of its downstream client proteins, including EGFR, MET, and AKT. Ultimately, this overcomes EGFR-T790M mutation-mediated resistance. These single or combined strategies targeting apoptosis pathways offer new multidimensional approaches against lung cancer drug resistance. They achieve this through precise regulation of ROS levels, autophagy processes, and protein degradation pathways. Future research must deeply explore their clinical translation potential. These findings not only deepen understanding of lung cancer cell death mechanisms but also lay a theoretical foundation for developing resistance-overcoming combination therapies.

### 3.2 Autophagy regulation therapy

Autophagy plays a dual role in lung cancer. It can promote tumor cell survival or induce cell death. Therefore, therapeutic strategies must selectively inhibit or enhance autophagic pathways based on specific mechanisms. For autophagy inhibition, 3-methyladenine (3-MA) blocks autophagosome formation. This action significantly enhances autophagic cell death induced by RBBP4 knockdown. Consequently, it suppresses NSCLC cell proliferation and promotes apoptosis. Preclinical studies show that combining RBBP4 silencing with 3-MA synergistically inhibits tumor growth in mouse xenografts (24). Conversely, autophagy induction strategies focus on natural compounds. Baicalin activates the lysosomal membrane channel MCOLN3. This activation disrupts Ca²^+^ homeostasis and causes lysosomal dysfunction. It blocks autophagic flux degradation. This leads to mitochondrial membrane potential depolarization and ROS accumulation. Ultimately, it suppresses tumor growth through autophagy-related apoptosis (16). Furthermore, polysaccharides from the polar yeast Cryptococcus heimaeyensis (CHEPS) activate the ROS/p38 and ROS/ERK signaling axes. They induce G2/M phase arrest and autophagy-dependent cell death. *In vivo*, CHEPS significantly inhibits orthotopic lung cancer progression without organ toxicity (59). Notably, combination therapies expand therapeutic applications. Berberine combined with EGFR-TKI overcomes TKI resistance by inducing autophagic degradation of EGFR protein (17). Polyphyllin VI (PPVI) simultaneously inhibits the PI3K/AKT/mTOR pathway and activates AMPK. This dual action synergistically induces both autophagy and apoptosis (60). Although these strategies show significant efficacy in preclinical models, clinical translation faces challenges. These include targeted delivery, dosage optimization, and drug resistance. Future research should explore synergistic mechanisms with immunotherapies like PD-1/PD-L1 inhibitors (61). In summary, precise modulation of autophagy advances lung cancer treatment. It moves from molecular mechanism exploration towards new clinical translation stages. This progress offers novel therapeutic strategies to overcome treatment resistance and improve prognosis.

### 3.3 Immunogenic cell death to enhance immunotherapy

**Figure 2:**
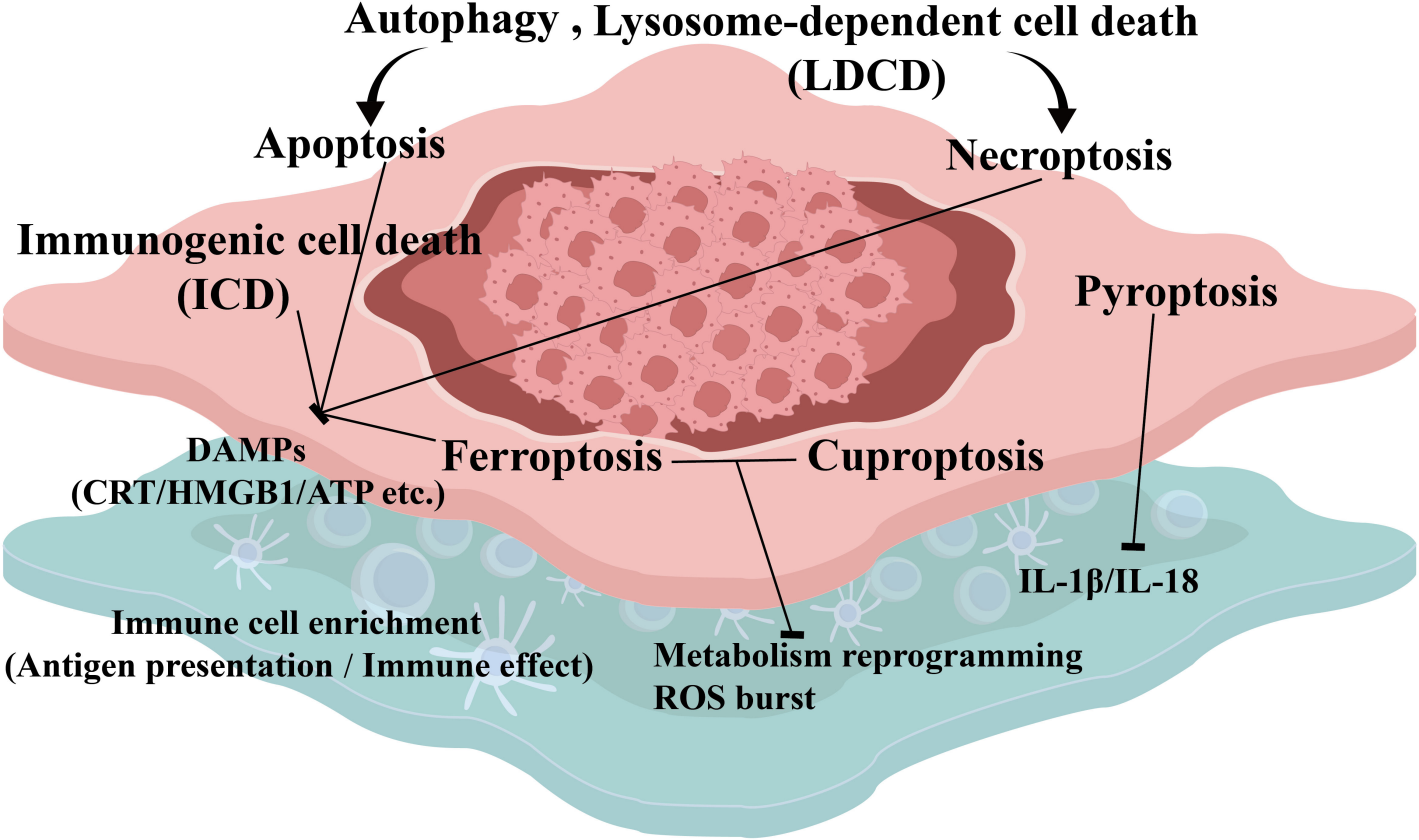
Interaction between RCD pathways and the tumor immune microenvironment. This figure illustrates how eight RCD pathways remodel the tumor immune microenvironment through key signaling molecules. Activation of distinct RCD pathways—such as pyroptosis, necroptosis, ferroptosis, and immunogenic cell death (ICD)—triggers tumor metabolic reprogramming and the release of numerous immunostimulatory signals (e.g., DAMPs: CRT, HMGB1, ATP; and proinflammatory cytokines: IL-1β, IL-18). These signaling molecules serve as bridging factors that ultimately drive tumor immune reprogramming, as depicted in the lower part of the figure: on one hand, they recruit and activate antigen-presenting cells (DCs and T cells); on the other hand, they enhance the infiltration and function of effector immune cells (cytotoxic T cells and NK cells). Consequently, the immunosuppressive state is reversed into an immunostimulatory state, collectively coordinating antitumor immune responses and ultimately leading to the reprogramming of the tumor immune microenvironment.

ICD activates anti-tumor immune responses by releasing DAMPs. This has become a key strategy to enhance lung cancer immunotherapy. Chemical inducers like (-)-guaiol significantly induce ICD in NSCLC (51). Its mechanism involves apoptosis and autophagy pathways mediating DAMP release. Released DAMPs include ATP and HMGB1. These promote dendritic cell (DC) activation and T-cell infiltration while suppressing tumor growth. Vaccination experiments confirm this induction provides long-term immune protection (51). Given ICD’s immune-activating potential its combination with ICIs shows significant synergistic prospects. For more precise guidance of combination therapy studies indicate PD-L1 blood markers predict ICI efficacy dynamically. These markers include soluble PD-L1 (sPD-L1) PD-L1-positive circulating tumor cells (PD-L1^+^ CTCs) and exosomal PD-L1 (exoPD-L1). High pre-treatment sPD-L1 or exoPD-L1 levels predict poor prognosis. Conversely post-treatment sPD-L1 downregulation or detected PD-L1^+^ CTCs correlate with significantly prolonged progression-free survival (PFS) and overall survival (OS) (19). Further optimizing patient selection and personalized treatment a prognostic model based on LDCD-related genes effectively distinguish NSCLC immune microenvironment features. Specifically low-risk group patients exhibit higher immune cell infiltration levels and longer survival. This provides a strong molecular subtyping basis for personalized immunotherapy in these patients (2). Therefore, targeting ICD induction not only directly enhances tumor immunogenicity but also overcomes immune resistance through biomarker-guided combination strategies (**Figure 2**). This opens a highly promising new pathway for lung cancer therapy. In conclusion deeply understanding and utilizing ICD and its related biomarkers is vital for achieving precision and high efficiency in lung cancer immunotherapy.

### 3.4 Targeting ferroptosis and necroptosis

Ferroptosis and necroptosis represent novel programmed cell death pathways. They show significant potential in targeted therapy for NSCLC. These pathways directly kill tumor cells. They also remodel the tumor immune microenvironment. This offers new directions for combination therapies (5, 8). Ferroptosis inducers hold broad application prospects. For instance, the thioredoxin reductase 1 (TrxR1) inhibitor Auranofin triggers ferroptosis. It does this through ROS accumulation and lipid peroxidation. This process also involves DNA damage. It shows particular sensitivity in p53-mutant NSCLC cells (8). Notably, Auranofin enhances NK cell cytotoxicity. It promotes ICD. This manifests as upregulated DAMPs. Examples include CRT exposure and ATP release (8). Furthermore, combining ferroptosis inducers with ICIs effectively overcomes resistance (55). This provides new strategies for patients unresponsive to PD-1/PD-L1 therapy.

Meanwhile, research on necroptosis agonists has progressed. Their core mechanism involves regulating key gene expression. Transmembrane protein 100 (TMEM100) shows low expression in NSCLC. Its loss induces autophagy-dependent necroptosis by inhibiting the PI3K/AKT pathway (5). Clinical studies confirm that low TMEM100 expression correlates significantly with advanced TNM stage. It also links to lymph node metastasis and poor prognosis. *In vivo* experiments demonstrate that TMEM100-targeted siRNA effectively suppresses tumor growth. It also upregulates necroptosis-associated protein levels (5). Additionally, small molecule agonists targeting receptor-interacting protein kinase (RIPK) induce necroptosis synergistically. Agents like TMPyP4 and thymoquinone work by disrupting lysosomal function and calcium ion balance. However, their selectivity requires further optimization (62, 63). In summary, exploring the molecular mechanisms of ferroptosis and necroptosis is crucial. Understanding their interplay with the immune system is equally important. This will lay a solid foundation for developing more effective personalized combination therapies for NSCLC.

### 3.5 Targeted pyroptosis and cuproptosis

Targeting pyroptosis and cuproptosis offers a dual strategy. This approach directly induces tumor cell death. It also synergistically enhances antitumor immunity through immunogenic effects. This opens new directions for precision immunotherapy in lung cancer.

Targeting pyroptosis has emerged as a novel strategy in lung cancer immunotherapy. For example, studies show the natural compound (-)-Guaiol triggers ICD. This activates antitumor immunity in NSCLC. The mechanism involves releasing pro-inflammatory factors like HMGB1 and ATP. It activates dendritic cells and increases T cell infiltration. Consequently, it significantly suppresses tumor growth (51). Another study reveals sea hare hydrolysates (SHH) work differently. SHH induces caspase-1-mediated pyroptosis/necroptosis in human lung cancer A549 cells. This occurs by inhibiting the STAT3 pathway. Key features include mitochondrial membrane potential collapse, ROS accumulation, and increased IL-1β secretion. This process remodels the tumor immune microenvironment (54). Importantly, potent pro-inflammatory factors released during pyroptosis enhance ICI efficacy. This provides a solid theoretical basis for combination therapy. Such therapy combines pyroptosis induction with immune checkpoint blockade. These findings collectively position pyroptosis targeting as a promising pathway for improving lung cancer immunotherapy outcomes.

Cuproptosis represents a novel form of regulated cell death. It shows promise for lung cancer treatment. Studies show an iridium (III) complex (Ir1) targets the endoplasmic reticulum. This induces endoplasmic reticulum stress. It also triggers ROS bursts (64). Consequently, it promotes CRT exposure on the cell surface. It also causes extracellular release of HMGB1 and ATP (64). This pathway activates CD8^+^ T-cell responses. Additionally, it depletes inhibitory regulatory T cells (Tregs). Therefore, it establishes and sustains long-lasting antitumor immunity (8). Simultaneously, auranofin acts as a thioredoxin reductase (TrxR) inhibitor. It disrupts intracellular copper ion homeostasis. This disruption induces ROS-dependent ferroptosis or apoptosis (8). Furthermore, dying cells release DAMPs. These DAMPs promote dendritic cell maturation. They also enhance NK cell-mediated cytotoxicity (65). Collectively, these findings highlight a key role. Imbalanced metal ion homeostasis, particularly copper, is crucial. It triggers immunogenic cell death forms like cuproptosis, ferroptosis, and apoptosis. It also drives subsequent antitumor immune responses. This provides vital theoretical support. It aids in developing new anti-lung cancer immunotherapies. These therapies would target metal ion homeostasis regulation.

### 3.6 Overcoming resistance to targeted therapy

**Figure 3:**
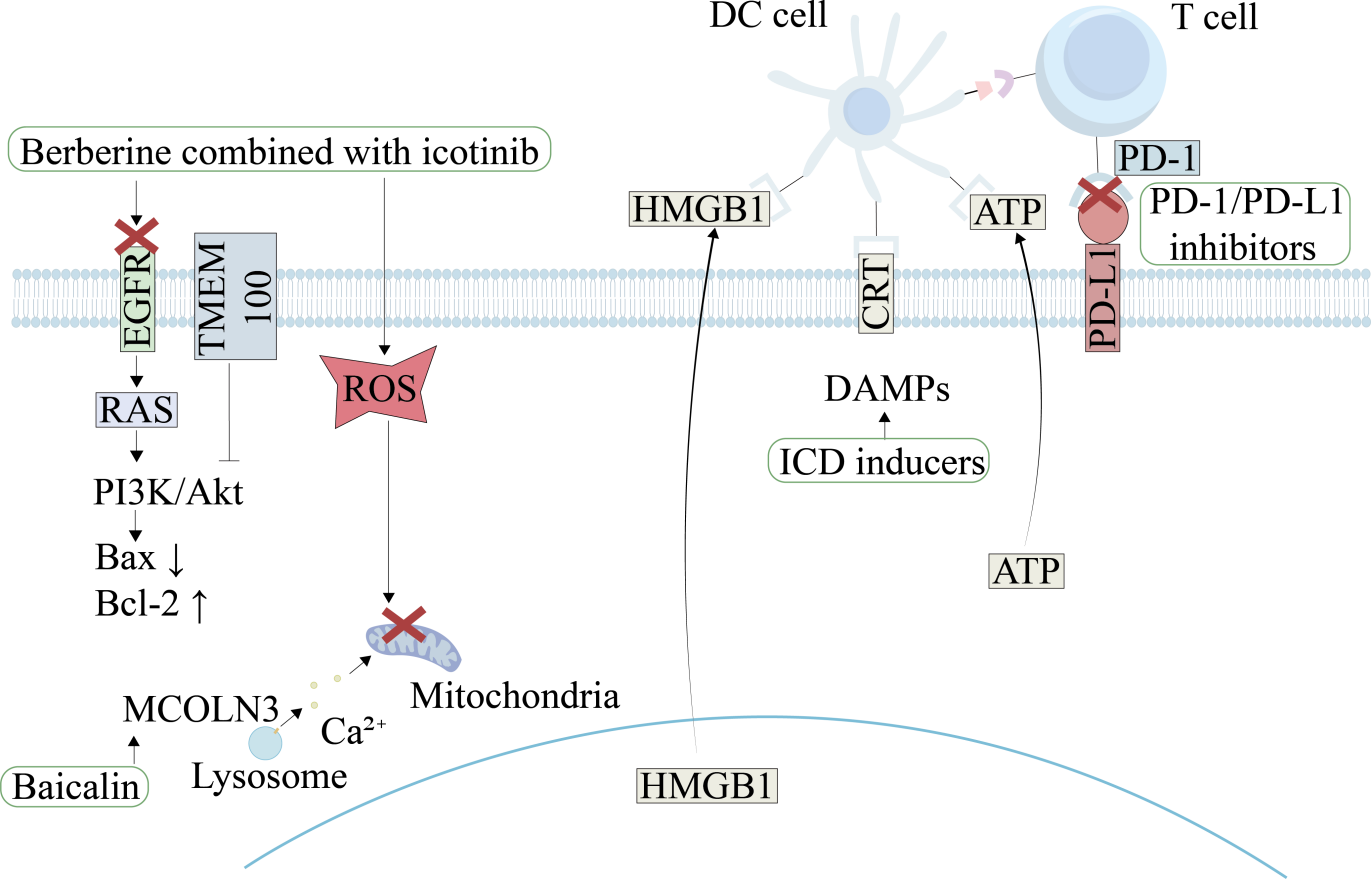
Targeting RCD pathways to overcome therapeutic resistance in NSCLC. This figure illustrates therapeutic strategies for reversing drug resistance by modulating RCD based on molecular mechanisms. Berberine combined with icotinib restores apoptotic susceptibility via EGFR degradation and ROS accumulation; baicalin triggers mitochondrial apoptosis by activating the MCOLN3 channel and disrupting lysosomal calcium homeostasis. Activation of TMEM100 can inhibit the PI3K/AKT pathway to induce both necroptosis and autophagy. Furthermore, ICD inducers such as (−)-Guaiol, when combined with PD-1/PD-L1 inhibitors, promote the release of DAMPs and significantly enhance T-cell immune responses.

To overcome resistance in lung cancer targeted therapy, researchers propose innovative intervention strategies. These focus on targeting metabolic vulnerabilities or epigenetic regulation pathways in resistant cells (**Figure 3**). For metabolic intervention, osimertinib-resistant NSCLC cells exhibit significant metabolic reprogramming. They show heightened dependence on mitochondrial metabolism pathways (6). Resistant cells sustain energy supply by enhancing glucose/glutamine uptake and utilization efficiency. Studies demonstrate that inhibiting glucose/pyruvate input into mitochondrial respiration or glutamine conversion to glutamate effectively suppresses resistant cell proliferation and promotes cell death (6). In epigenetic regulation, the natural compound evodiamine reverses cisplatin resistance. This mechanism involves significant ROS elevation. Evodiamine downregulates SOX9 expression and blocks the SOX9/β-catenin signaling axis. Consequently, it restores tumor cell sensitivity to cisplatin (66). *In vitro* and *in vivo* experiments further confirm that evodiamine combined with cisplatin synergistically induces autophagy-dependent cell death. This combination significantly inhibits tumor growth (66). Targeting metabolic reprogramming or epigenetic pathways thus offers novel directions with translational potential against lung cancer drug resistance. These strategies show translational potential for overcoming resistance. Their development and validation may offer more effective treatment options for patients with resistant lung cancer.

## 4 Biomarkers and treatment response prediction

**Figure 4:**
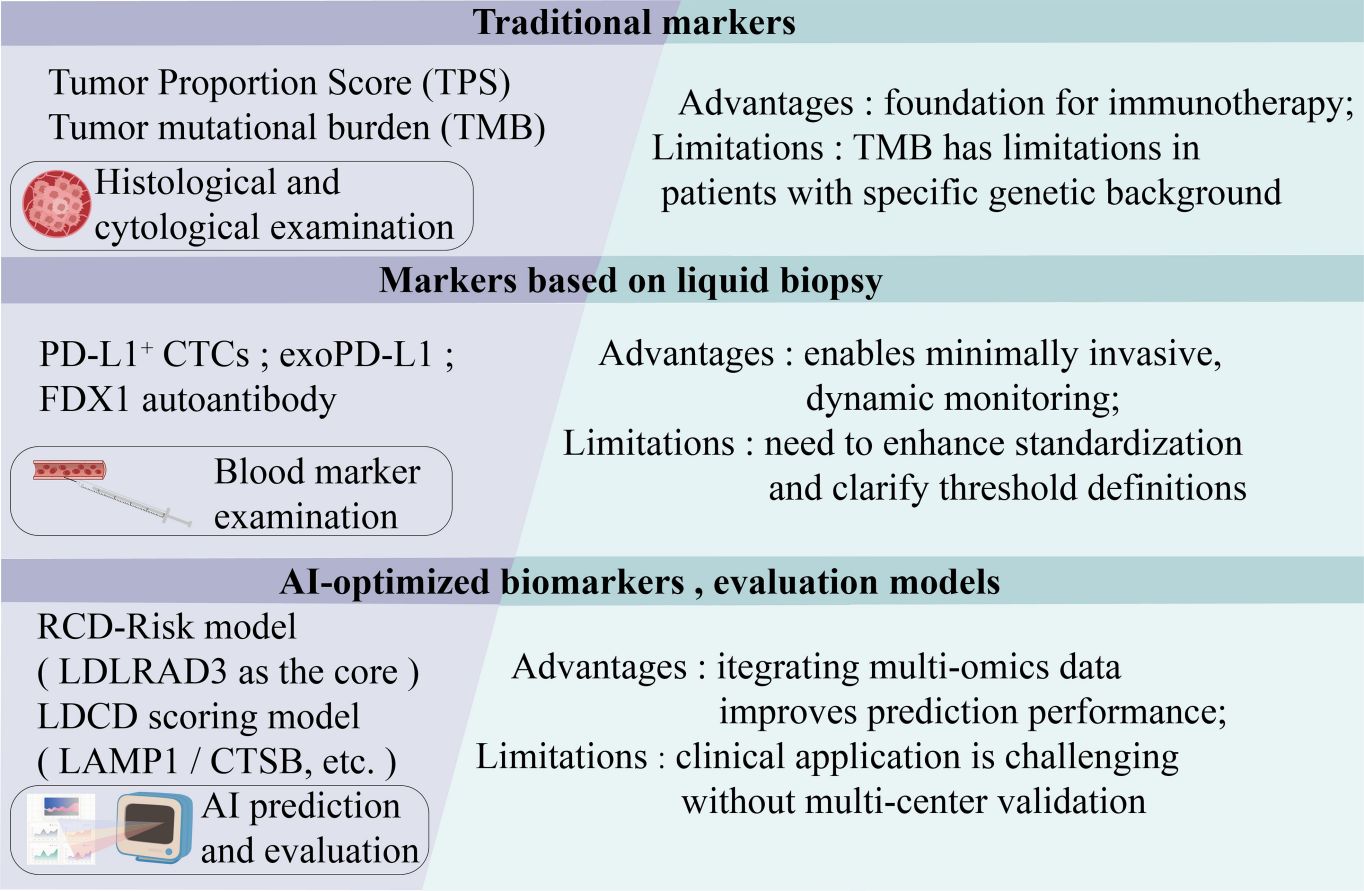
Predictive biomarker system for precision therapy in NSCLC. This figure illustrates a predictive biomarker system for precision therapy in lung cancer, encompassing three categories: conventional, liquid biopsy, and AI-optimized biomarkers. Among conventional biomarkers, PD-L1 (assessed by tissue tumor proportion score [TPS]) and tumor mutational burden (TMB) have established a molecular basis for predicting response to immunotherapy in clinical practice; however, they are constrained by limitations such as inconsistent detection methods and predictive blind spots. Liquid biopsy biomarkers (e.g., PD-L1^+^ CTCs and exoPD-L1) allow minimally invasive dynamic monitoring, yet standardization and threshold definition remain challenging. AI-optimized models (e.g., the LDCD score and RCD-Risk) significantly enhance predictive performance by integrating multi-omics data but face issues such as insufficient multi-center validation and barriers to clinical application. In summary, conventional biomarkers provide a foundational framework, liquid biopsy enables dynamic monitoring, and AI-driven models offer high-dimensional integration and precise predictive capabilities—collectively advancing precision therapy for NSCLC into a new era of multidimensional and intelligent decision-making.

Targeting RCD pathways exhibits variable efficacy in lung cancer, and immune checkpoint inhibitors (ICIs) frequently encounter clinical resistance. Therefore, reliable biomarkers for patient stratification are essential to guide personalized precision therapy. This section reviews key predictive biomarkers for ICI efficacy, focusing on the established role of programmed death-ligand 1 (PD-L1) expression and tumor mutational burden (TMB), and explores the innovative potential of predictive models based on RCD gene signatures (**Figure 4**).

### 4.1 PD-L1 expression

PD-L1 expression remains a cornerstone predictive biomarker for ICI efficacy in NSCLC (67, 68). High tissue expression (TPS ≥50%) is clinically used to identify patients likely to benefit. Importantly, PD-L1 assessment on cytological specimens shows high concordance with tissue biopsies, supporting its utility across sample types (21, 69). Beyond static tissue analysis, dynamic monitoring of circulating biomarkers provides additional prognostic insight. Elevated pre-treatment levels of soluble PD-L1 (sPD-L1) correlate with primary resistance and worse survival (70), while circulating exosomal PD-L1 (exoPD-L1) and PD-L1-positive circulating tumor cells offer complementary information on disease burden and potential treatment benefit (19). Thus, integrating static tissue PD-L1 with dynamic liquid biopsy markers enables a more nuanced prediction of ICI response. It is noteworthy that the expression and functional role of PD-L1 are dynamically shaped by the tumor immune microenvironment, which is, in turn, profoundly influenced by ongoing cell death processes.

### 4.2 Tumor mutation burden

Tumor mutational burden (TMB), quantifiable via targeted next-generation sequencing, serves as an independent biomarker predictive of ICI response (71). Patients with high TMB show significantly improved durable clinical benefit and progression-free survival following ICI treatment. TMB and PD-L1 provide complementary information, and their combined assessment can enhance patient selection. However, specific genomic features, such as a high fraction of copy number-altered genome or the presence of EGFR/STK11 variants, are associated with reduced ICI benefit, underscoring the complexity of the tumor-immune interface (71). As a key quantitative measure of immune microenvironment activation, TMB establishes a molecular foundation for precision immunotherapy. However, the immunogenic potential of these mutations, and thus their translation into effective anti-tumor immunity, may depend on the cell death pathways that are activated in response to genomic stress.

### 4.3 Integrating RCD activity with conventional and novel biomarkers

Molecular subtyping based on RCD-related genes represents a novel and integrative approach to predicting tumor immune features and therapy response. Models such as the RCD-Risk score and the LDCD signature effectively stratify patient prognosis and correlate with distinct immune microenvironment characteristics, functionally quantifying the activity of specific cell death pathways (2, 72). Critically, the immunogenic context sculpted by RCD pathways may interact with and refine the predictive power of conventional biomarkers. For instance, a tumor with high RCD activity (e.g., evident from a high LDCD score) might exhibit enhanced antigen presentation and T-cell infiltration, potentially augmenting the efficacy of PD-1/PD-L1 blockade even in cases with moderate PD-L1 expression. Conversely, resistance to immunogenic RCD could contribute to an immunosuppressive milieu, limiting ICI benefit despite high TMB. Therefore, integrating RCD activity signatures with established markers like PD-L1 and TMB could enable more nuanced, multi-dimensional patient stratification, better identifying candidates for combinations of RCD-targeting agents and immunotherapy.

## 5 Future research perspectives

While significant advances have been made in therapeutic strategies targeting RCD pathways, core bottlenecks persist including drug resistance, relapse, and insufficient remodeling of the immune microenvironment. Overcoming these limitations urgently requires focus on the following key directions.

### 5.1 Exploration of novel cell death mechanisms

While the roles of apoptosis, autophagy, and ICD in lung cancer are relatively well-defined, research on novel mechanisms like necroptosis, ferroptosis, and methionine restriction-induced cell death remains nascent. For example, studies show DHA enhances cisplatin’s lethality against NSCLC. This occurs by inhibiting prostaglandin G/H synthase 1 (PTGS1) expression and activating the ROS-endoplasmic reticulum stress-JNK/p38 MAPK signaling axis (55). Furthermore, prognostic models built using LDCD-associated genes effectively predict immune microenvironment features in NSCLC (2). Additionally, RBBP4 was found to influence tumor immune infiltration by regulating autophagy gene expression (24). Therefore, deeply analyzing the molecular regulation of these novel cell death pathways will provide new therapeutic targets for lung cancer. These discoveries expand our understanding of lung cancer cell death complexity and also signal future directions for innovating precision treatment strategies.

### 5.2 Synergistic regulation of multiple death pathways

Activating multiple RCD pathways simultaneously represents a novel strategy to overcome drug resistance. This is because triggering only one death pathway often leads to treatment resistance. For instance, an iridium (III) complex localizes to the endoplasmic reticulum. It induces ICD markers like CRT exposure and HMGB1 release. Concurrently, it also triggers apoptosis (53). Another study showed that the antibody-drug conjugate luveltamab tazevibulin induces ICD. It also enhances CD8^+^ T cell infiltration. Combining it with immune checkpoint inhibitors significantly suppresses tumor growth (73). Among natural compounds, (-)-guaiol induces both apoptosis and autophagy. It further triggers ICD (28). Separately, baicalin activates the MCOLN3 channel. This blocks autophagic flux and simultaneously induces mitochondrial apoptosis (16). Collectively, these findings indicate that developing inducers capable of effectively activating dual RCD pathways holds great promise. Such agents could break compensatory resistance barriers in lung cancer therapy. They offer potential for more durable antitumor solutions clinically.

### 5.3 Overcoming the immunotherapy bottleneck

Despite advances with immune checkpoint inhibitors (ICIs) in NSCLC therapy, patients harboring EGFR mutations typically show low response rates. Consequently, exploring combination strategies is essential for optimization. A meta-analysis demonstrated that for patients resistant to tyrosine kinase inhibitors (TKIs), combining PD-L1 inhibitors with VEGF inhibitors and chemotherapy significantly improved progression-free survival (PFS). However, this combination offered relatively limited overall survival (OS) benefit (74). Furthermore, baseline corticosteroid use before treatment has been confirmed to diminish the efficacy of PD-L1 blockade (75). Additionally, higher pre-treatment levels of serum soluble PD-L1 (sPD-L1) and exosomal PD-L1 (exoPD-L1) predict poorer responses to ICI therapy (19). Overall, targeting the immunosuppressive microenvironment combined with strategies inducing regulated cell death represents a critical research direction for enhancing response rates in these therapy-resistant patients.

### 5.4 Leveraging advanced computational tools to decipher RCD complexity and guide translation

Advanced computational tools are pivotal for navigating the complexity of RCD pathways and accelerating their clinical translation. By integrating multi-omics data (e.g., single-cell and spatial transcriptomics), these methods can decode the heterogeneous activation and crosstalk of distinct RCD modes within the tumor microenvironment, as evidenced by prognostic models based on specific RCD-related signatures such as the LDCD score (2). To build robust predictive models for patient stratification, computational analysis is increasingly fused with dynamic, multi-modal data streams. These include AI-enhanced digital pathology for precise biomarker quantification and longitudinal immune monitoring via techniques such as T-cell receptor sequencing, which respectively improve diagnostic accuracy and enable dynamic response assessment (76, 77). Such integrative models are essential for identifying patients most likely to benefit from specific RCD-inducing therapies or rational drug combinations. Moreover, computational approaches streamline the discovery of novel RCD-targeting agents and inform the design of biomarker-enriched clinical trials, thereby bridging fundamental RCD biology and precision therapeutic intervention.

### 5.5 Clinical translation challenges

The clinical translation of therapeutic strategies targeting RCD faces several interconnected challenges that must be addressed to realize their full potential in lung cancer treatment. Key issues include the management of combination therapy toxicity, the optimization of dosing schedules, and the standardization of predictive biomarkers. For instance, while combining immune checkpoint inhibitors with chemotherapy can improve progression-free survival in certain patient subsets such as those with EGFR-mutant NSCLC, the overall survival benefit often remains modest and is accompanied by significantly increased toxicity (74). PD-L1 expression remains a pivotal biomarker, yet its assessment requires further harmonization to ensure consistency across different specimen types (21). Additionally, the prognostic role of PD-1/PD-L1 in patients with rare EGFR mutations awaits validation in larger cohorts (78). Promisingly, adjunct approaches such as nutritional interventions have shown potential to modulate metabolism and reverse immunotherapy resistance in hypermetabolic patients, highlighting novel avenues to overcome treatment barriers (79). Future progress will hinge on deep multidisciplinary collaboration that integrates fundamental RCD biology with clinical innovation—including the use of advanced computational tools to decipher pathway crosstalk and guide patient stratification. Ultimately, overcoming these translational bottlenecks through well-designed prospective trials and iterative biomarker development is essential to maximize the therapeutic impact of RCD-targeted therapies in lung cancer.

## Glossary

AIM2: Absent in melanoma 2
AKT: Protein kinase B
APC: Antigen-presenting cell
ASC: Apoptosis-associated speck-like protein containing a CARD
ATOX1: Antioxidant 1 copper chaperone
ATP: Adenosine triphosphate
AURKA: Aurora kinase A
BAX: BCL2-associated X protein
BCL-2: B-cell lymphoma 2
CAF: Cancer-associated fibroblast
CASP1: Caspase-1
CRT: Calreticulin
CTSB: Cathepsin B
CuB: Cucurbitacin B
CXCL5: C-X-C motif chemokine ligand 5
DAMP: Damage-associated molecular pattern
DC: Dendritic cell
DHA: Dihydroartemisinin
DLAT: Dihydrolipoamide S-acetyltransferase
DLST: Dihydrolipoamide S-succinyltransferase
EGFR: Epidermal Growth Factor Receptor
ERK: Extracellular signal-regulated kinase
FDX1: Ferredoxin 1
GSDMD: Gasdermin D
GSDME: Gasdermin E
GPX4: Glutathione peroxidase 4
HMGB1: High mobility group box 1
ICD: Immunogenic cell death
ICI: Immune checkpoint inhibitor
IFN-γ: Interferon-gamma
IL-1β: Interleukin-1 beta
IL-12: Interleukin-12
IL-18: Interleukin-18
JNK: c-Jun N-terminal kinase
LAMP1: Lysosomal-associated membrane protein 1
LDCD: Lysosome-dependent cell death
LIPT1: Lipoyltransferase 1
LMP: Lysosomal membrane permeabilization
lncRNA: Long non-coding RNA
MAPK: Mitogen-activated protein kinase
MCOLN3: Mucolipin 3
MLKL: Mixed lineage kinase domain-like protein
mTOR: Mammalian target of rapamycin
NF-κB: Nuclear factor kappa-light-chain-enhancer of activated B cells
NK cell: Natural killer cell
NLRP3: NLR family pyrin domain containing 3
NSCLC: Non-small cell lung cancer
OS: Overall survival
OV: Oncolytic virus
PD-1: Programmed cell death protein 1
PD-L1: Programmed death-ligand 1
PFS: Progression-free survival
PI3K: Phosphatidylinositol 3-kinase
RBBP4: Retinoblastoma-binding protein 4
RCD: Regulated cell death
RIPK1: Receptor-interacting serine/threonine-protein kinase 1
RIPK3: Receptor-interacting serine/threonine-protein kinase 3
ROS: Reactive oxygen species
SCLC: Small cell lung cancer
STAT3: Signal transducer and activator of transcription 3
TCA cycle: Tricarboxylic acid cycle
TCR: T-cell receptor
Th1: T helper 1
TIL: Tumor-infiltrating lymphocyte
TLR4: Toll-like receptor 4
TMEM100: Transmembrane protein 100
TMB: Tumor mutational burden
TNF: Tumor necrosis factor
TKI: Tyrosine kinase inhibitor
TNM: Tumor, Node, Metastasis (staging system)
Treg: Regulatory T cell
TrxR1: Thioredoxin reductase 1
VEGF: Vascular endothelial growth factor
